# Wnt Signaling in Cell Motility and Invasion: Drawing Parallels between Development and Cancer

**DOI:** 10.3390/cancers8090080

**Published:** 2016-08-29

**Authors:** Alanna E. Sedgwick, Crislyn D’Souza-Schorey

**Affiliations:** Department of Biological Sciences, University of Notre Dame, Notre Dame, IN 46656, USA; Alanna.E.Sedgwick.1@nd.edu

**Keywords:** Wnts, cell motility, cell invasion, cancer, development, small GTP-binding proteins

## Abstract

The importance of canonical and non-canonical Wnt signal transduction cascades in embryonic development and tissue homeostasis is well recognized. The aberrant activation of these pathways in the adult leads to abnormal cellular behaviors, and tumor progression is frequently a consequence. Here we discuss recent findings and analogies between Wnt signaling in developmental processes and tumor progression, with a particular focus on cell motility and matrix invasion and highlight the roles of the ARF (ADP-Ribosylation Factor) and Rho-family small GTP-binding proteins. Wnt-regulated signal transduction from cell surface receptors, signaling endosomes and/or extracellular vesicles has the potential to profoundly influence cell movement, matrix degradation and paracrine signaling in both development and disease.

## 1. Introduction

The loss of normal cell polarity and adhesion, along with the acquisition of motility and invasiveness, are fundamental steps during tumor progression and metastasis. The process of metastasis, wherein cells disseminate from a tumor and grow at distant locations, remains the largest contributor to cancer mortality [1,2]. The dysregulation of many signaling pathways, including Wnt signaling, contribute to this behavior. Wnts are well-characterized for critical functions during normal embryogenesis and tissue homeostasis, regulating processes such as cell motility, adhesion, invasion, tissue patterning, and proliferation [3,4]. However, aberrant Wnt signaling in the adult frequently leads to abnormal cellular behaviors, progressing to the onset of disease. Many aspects of Wnt signaling have been reviewed extensively in the literature, and here we describe known and predicted parallels between Wnt-mediated regulation of normal embryonic and tissue homeostatic behavior, and the abnormal activation of these pathways in cancer progression, with particular focus on tumor cell motility and invasion. Wnt-mediated regulation of the dynamics of signaling endosomes, extracellular vesicles, and invadopodia have the capacity to impact tumor cell invasion and extracellular matrix degradation. We discuss recent findings on the roles of canonical and non-canonical Wnt pathways and small GTPase-mediated signaling in the modulation of these processes, and outline unresolved gaps in the field that merit further study.

## 2. Canonical and Non-Canonical Wnt Signaling—An Overview

There are currently nineteen Wnt ligands identified for both canonical and non-canonical signaling axes, with some ligands functioning through both pathways [5,6]. Wnt receptors LRP5 and LRP6, alongside the ten members of the frizzled (Fzd) family of G-protein-coupled receptors, mediate canonical signaling pathways [7,8]. ROR1, ROR2 (receptor tyrosine kinases), and RYK (receptor-like tyrosine kinase) function as alternative Wnt receptors in non-canonical signaling pathways, though this signal transduction may also modulate canonical signal transduction [5,9,10,11]. The large number of Wnt ligands and receptors potentially allows for great diversity in signaling outcomes [5].

**Figure 1:**
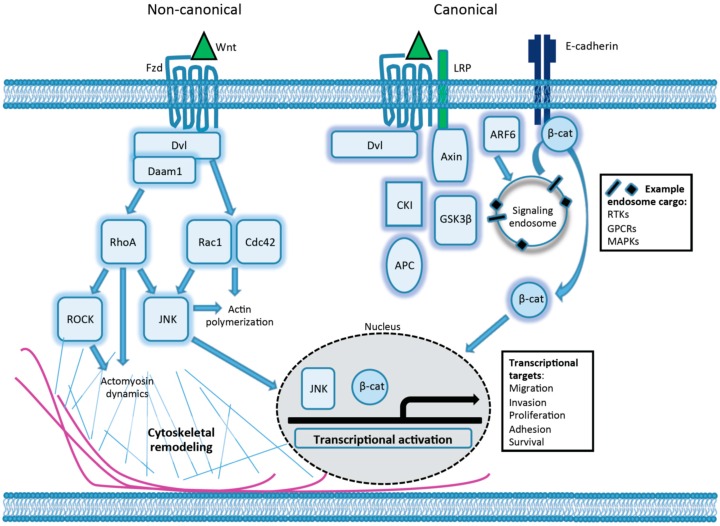
Canonical and non-canonical Wnt pathways influence cell migration and invasion. Wnt activation may prompt the activation of multiple downstream signaling pathways, selected examples of which are shown here. Canonical Wnt signal transduction results in the dissociation of the “destruction complex” and stabilization of cytoplasmic β-catenin, which then translocates to the nucleus to regulate the transcription of downstream targets. Canonical Wnt signaling has also been shown to stimulate ARF6 activity, which in turn promotes the internalization of transcriptionally active β-catenin from sites of cadherin-based adhesion. Non-canonical signaling cascades promote the activation of the small GTPases Rac1, RhoA, and Cdc42 to promote the cytoskeletal remodeling needed for cell invasion and migration, as well as JNK to regulate additional transcriptional targets.

The cellular processes modulated by Wnts range from stem cell self-renewal to cell motility, and are mediated by transcriptional activation as well as through direct effects on cytoplasmic targets [3,12]. β-catenin is a critical component in many Wnt pathways, and functions as both a cell-cell adhesion protein and also an intracellular signaling molecule [13,14]. Cytoplasmic β-catenin is typically degraded in the proteasome following phosphorylation by the destruction complex, which is composed of adenomatous polyposis coli (APC), Axin 1/2, casein kinase I (CKI), and glycogen synthase kinase 3β (GSK3β), and subsequent ubiquitination by β-transducin repeat-containing protein (β-Trcp). Wnts are the best known inhibitors of this degradation. Wnt ligands activate canonical signaling by binding Fzd and LRP5/6 receptors at the cell surface, and LRP phosphorylation mediates the recruitment of Axin and its subsequent inactivation, prompting the dissociation of the destruction complex and freeing β-catenin to translocate to the nucleus [4,15] (Figure 1). Nuclear β-catenin acts as a transcriptional co-activator for a variety of downstream targets of the TCF/LEF family of transcription factors, affecting the transcription of target genes which regulate a large and diverse set of cellular processes including apoptosis, metabolism, proliferation, motility, cell cycle progression, and differentiation [16,17].

Non-canonical Wnt signaling, which is independent of β-catenin transcriptional activity, encompasses multiple signaling cascades which signal through Fzd; Fzd alternative receptors ROR1, ROR2, or RYK; or possibly through Fzd with ROR or RYK as co-receptors [5,7,9,10,18]. Non-canonical signal transduction engages several downstream effectors, including calmodulin/calcium, protein kinase C (PKC), heterotrimeric G proteins, Src, JNK, and multiple small GTPases [19].

Of the non-canonical pathways, the planar cell polarity (PCP) pathway is the best characterized, with influence over a variety of developmental and disease processes [20,21,22]. Of the nineteen identified Wnt ligands, seven are characterized to work in non-canonical pathways [23], with Wnts 4, 5a, and 11 as the best characterized to influence PCP signaling. PCP signaling is critical for polarized cell movement and the uniform alignment of cell polarity and patterning during tissue formation [12,21,24,25,26,27,28]. As such, PCP signaling is key during neural crest migration [27], when cells must migrate from the dorsum of the neural tube following an epithelial-to-mesenchymal transition and travel to their final destination where they will differentiate into a wide variety of cells ranging from cartilage to neurons to melanocytes [29]. This migration occurs in highly polarized cell streams which are shaped by the presence of negative signals from the surrounding environment [27]. PCP signaling has also been shown to regulate additional, diverse developmental behaviors, including vertebrate gastrulation [24,28], neural tube closure [25], establishment of normal ciliary function [26], and the planar orientation of cell division [24].

## 3. Wnt Signaling in Cancer—An Overview

While canonical and non-canonical Wnt regulation of activities such as differentiation, adhesion, cell morphology, and motility are critical for normal development in the embryo [3,13], aberrant signaling through Wnt pathways can promote cancer development and progression [13,19,22,30,31]. A role for Wnt in cancer was initially identified when the tumorigenic mouse mammary tumor virus (MMTV) was frequently found to integrate into a particular region of the genome, then named MMTV int1 [32], and later recognized to encode the first identified Wnt, later to be named as Wnt1 [33]. A strong association of Wnt signaling in human cancer was identified when a correlation was noted between mutations of the β-catenin regulatory protein APC and familial adenomatous polyposis, which confers a greatly increased incidence of colorectal cancer [13,34,35]. Mutations of APC have now been characterized in approximately 80 percent of sporadic colon tumors [36,37], and less frequently in cancer of other tissues such as breast, stomach, and lung [19,38,39,40]. Mutations in β-catenin itself are also frequently found in tumors of the liver, pituitary gland, endometrium, pancreas, and certain ovarian cancers, with a large number of mutations in regions which prevent its degradation [19,41,42]. Mutation of the destruction complex protein Axin is also common, noted in a variety of cancers including medulloblastoma, melanoma, adenoid cystic carcinoma, and hepatic cancers [19,43].

It is important to note that Wnts do not always promote tumor progression, however, and may have oncogenic or tumor suppressive roles depending on the cellular context. An example of this is Wnt5a, which has been shown to correlate with poor prognosis and promote tumor cell invasion in melanoma, glioma, pancreatic, prostate and gastric cancer [44,45,46,47,48,49], however in breast cancer, Wnt5a has been better characterized as a tumor suppressor [50,51,52].

## 4. Small GTPases in Wnt-Regulated Cell Motility and Invasion: Parallels between Development and Cancer

### 4.1. Conserved Cell Behavior: Motility and Invasion in Development and Disease

There are many similarities evident between the normal behaviors of cells during embryogenesis, and tumor cells during invasion and metastasis. Genetic mutations that subvert embryonic signaling modules are frequently characterized in cancer development. As an example, many parallels exist between the behavior of migrating neural crest cells in a developing embryo and invasive tumor cells migrating during the course of metastasis to a secondary site. Melanoma, the most frequently fatal skin cancer, is one such example [53]. One of the characteristics of advanced melanoma is aggressive tissue invasion [54], reminiscent of the activity that neural crest-derived melanocytes display during development, as cells must move through the dermis to colonize the skin and hair follicles [29].

The regulation of such cell motility and invasion is complex and multifaceted, and cells can utilize qualitatively different modes of movement to traverse distances [55,56]. These different motile behaviors are observed during embryogenesis and tissue remodeling, and tumor cells may exploit similar mechanisms. In cells with a rounded or amoeboid morphology, actomyosin at the cell cortex contracts in the direction of the desired flow. On the other hand, cells adopting a flattened or mesenchymal morphology generally form a leading edge that extends actin-rich protrusions such as lamellipodia, alongside adhesive interactions with the substratum, and followed by retraction of the contractile cell rear to achieve cellular movement [56,57]. The cytoskeletal organization required for amoeboid and mesenchymal movement is regulated by the selective activation or suppression of the Rho and ARF families of the Ras superfamily of small GTPases, as described further below. Both canonical and non-canonical Wnt signaling cascades intersect with these regulatory molecules to influence motile behavior [27,31,58,59] (Figure 1).

### 4.2. Roles for Rac1 and RhoA in Wnt-Mediated Cell Motility and Invasion

Intracellular signaling mediated by the small GTPases Rac1 and RhoA is pivotal in mediating Wnt activity in cell motility during development, regulating the formation of cell protrusions and directional migration [12,58]. Rac and Rho can directly influence actin rearrangements, as well as promote JNK activation to further alter cytoskeletal dynamics and gene transcription during several developmental processes [8,18]. In embryonic development, Rac1 activity has been characterized as important for canonical Wnt signaling downstream of Wnt3a and required for the translocation of β-catenin to the nucleus [60]. In this regard, mutation of the JNK2 phosphorylation sites of β-catenin at Ser191 and Ser605 abrogate the activation of Rac1. During neural crest migration, Rac1 is activated at the cell’s leading edge, promoting actin polymerization for cell protrusion, with RhoA activated at the retracting rear of the cell, to promote actomyosin contractility [27]. Inhibition of cell-cell contact upon collision of streaming cells is promoted by RhoA activity with a concomitant suppression of Rac1, to promote the retraction of cell protrusions [27].

Upon the binding of Wnt to a Fzd receptor, cytoplasmic Disheveled (Dvl) activates the formin Disheveled Associated Activator of Morphogenesis 1 (Daam1), facilitating the formation of a complex between Daam1, Dvl, and Rho, thereby facilitating Rho activation and subsequent Rho kinase activity [21]. To promote the formation of cell protrusions at the leading edge of the cell, Rac1 and JNK activity may also be enhanced by Dvl, to facilitate actin remodeling [21,61]. In the directional movement of neural crest cells in the Xenopus and zebrafish embryo, Rac1 and RhoA-mediated regulation of cell protrusions is also important. In this context, the proteoglycan Syndecan-4, which is required for directional migration of neural crest cells, downregulates Rac1 activation to control the direction of cell protrusion development [62]. In this process, PCP-mediated RhoA activity inhibits Rac1 in order to regulate directional migration.

During the course of development, embryonic cells adopt distinct morphologies to migrate during tissue organization, and this is also seen in adult tissues during healing and regeneration events [31,63,64]. An antagonistic relationship between Rac1 and RhoA signaling has been well-characterized for regulating mesenchymal versus amoeboid modes of motility [55,65], and an example of this behavior is seen during zebrafish gastrulation, when mesodermal cells must transition from an amoeboid motility to mesenchymal movement and then finally into polarized alignment [66]. This is mediated by RhoA, which promotes the inhibitory phosphorylation of myosin phosphatase to regulate acto-myosin protrusive activity. In the context of skeletal muscle tissue regeneration, the activation of Rho and JNK, believed to be through non-canonical Wnt signaling, is important for the blebbing movement utilized by satellite cells, the resident stem cell population in skeletal muscle [63]. Conversely, in the migration of fibroblasts and epithelial cells, a mesenchymal mode of movement, regulated by Rac1 through Wnt5a, has shown to be important [31].

**Figure 2:**
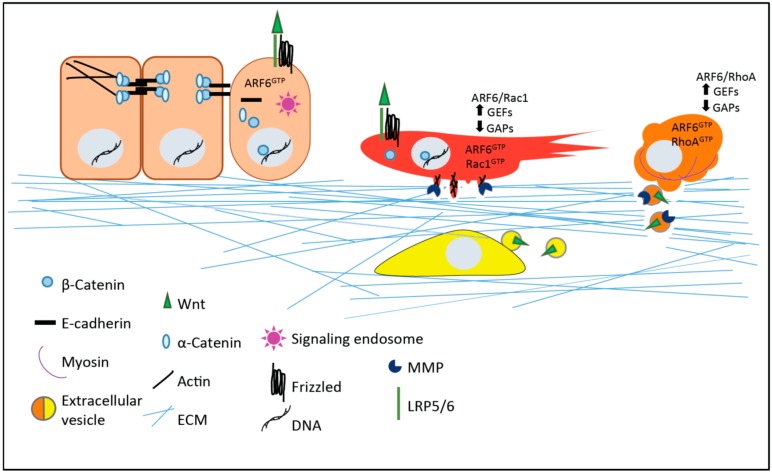
Wnt signaling impacts tumor progression and invasion. Wnt-regulated cellular changes include facilitating the dissociation and internalization of cadherin-based adhesions (cuboidal cells on the left) and the formation of invadopodia in migratory tumor cells (red). Wnt may also be included as cargo in extracellular vesicles released from amoeboid tumor cells (orange) as well as other cells such as fibroblasts in the tumor microenvironment (yellow). Small GTP-binding proteins of the ARF and Rho families, regulated by various GTPase activating proteins (GAPs) and guanine nucleotide exchange factors (GEFs), mediate many of these processes.

Amoeboid and mesenchymal modes of motility are also utilized by tumor cells as they navigate the extracellular environment. The alteration of cell morphology and motility is key during the course of cell invasion and metastasis, as cells encounter varied extracellular matrix barriers en route to a secondary site. Rac1 has a number of well-characterized functions in the movement of both normal and tumor cells, traditionally shown to be important for actin polymerization to drive lamellipodia-based movement and the formation of invadopodia to facilitate matrix proteolysis [57,67]. In tumor cells invading a rigid extracellular matrix environment, an upregulation of Rac1 activity and a concomitant downregulation of RhoA has been noted [57], similar to the signaling which has been observed during neural crest migration [62]. Tumor cells invading softer, more compliant extracellular matrix environments adopt rounded, amoeboid morphologies. These cells exhibit RhoA-mediated inactivation of myosin phosphatase, to promote the actomyosin-based contractility required for release of protease-rich invasive microvesicles from the cell surface [57], similar to the regulatory mechanism outlined above for cell motility during zebrafish gastrulation [66]. Both amoeboid and mesenchymal modes of invasion require upstream activation of the ARF6 small GTP-binding protein (described further below), that prompts Rac1 or RhoA activation depending on the compliance of the extracellular matrix [57] (Figure 2).

The activities of the Rho family proteins downstream of Wnts in cancer are wide-ranging and appear to be dependent on the cellular context. In a model of colorectal cancer, RhoA was found to function as a tumor suppressor, with its inactivation being required for canonical signaling downstream of Wnt3a to promote metastasis. Further reduced levels of RhoA were observed at metastatic sites when compared to primary lesions [68]. A Chinese hamster ovary cell line model of motility, however, found RhoA to be activated by Wnt3a, promoting actin reorganization and cell movement in concert with Dvl2 [69]. In an oral squamous cell carcinoma model, cells engineered to express β-catenin which lacks the entire GSK3β phosphorylation site showed cytoplasmic and nuclear β-catenin accumulation, and a corresponding increase in Rac1 and Cdc24 activation with the adoption of spindle-like elongated morphologies, loss of cell-cell contacts, and increased invasiveness [70]. During hepatocellular carcinoma progression, Rac1 activity has been shown to promote cell motility, and this can be antagonized by the activation of RhoA and Rho kinase downstream of Wnt activity [71]. In this instance, Wnt11 functions as a tumor suppressor by activating PKC signaling to phosphorylate β-catenin, resulting in reduced TCF-mediated transcriptional activity and decreased cell proliferation.

### 4.3. ARF6 and Wnt Signaling Regulate Tumor Cell Invasion and Signaling Endosome Formation

The small GTP-binding protein ARF6 regulates early endocytic membrane trafficking and rearrangements of the actin cytoskeleton, in part through its effects on phosphoinositide metabolism [72]. Mice homozygous for a null allele display embryonic and perinatal lethality beginning around mid-gestation, with impaired hepatic cord formation during liver development [73]. ARF6 is frequently overexpressed in cancers such as lung, breast, prostate, and colon [74,75,76,77], and its activation is associated with increased tissue invasion and increased formation of invadopodia and tumor-derived microvesicles to facilitate movement through the extracellular matrix [75,78,79,80,81].

Melanoma provides a prominent example for the role of Wnts and ARF6 in tumor invasion. A role for Wnt5a in melanoma progression through both canonical and non-canonical signal transduction has been demonstrated [46,48], which may be modulated by ARF signaling. Wnt5a, best characterized as a non-canonical signaling ligand, has been shown to promote melanoma invasion in a PKC-dependent manner, signaling through Fzd5 [48]. Additionally, canonical signaling through Wnt5a has been shown to promote ARF6 activation and subsequent melanoma invasion by stimulating β-catenin-mediated transcriptional activity [46]. In this instance, Wnt5a activates ARF6 by stimulating the activity of its exchange factor GEP100, through the receptors Fzd4 and LRP6. Blocking this pathway prevented lung metastasis in mice with melanoma xenograft tumors. In a separate study, the activities of ARF6 and ARF1 were shown to be important regulators of Wnt3a-mediated canonical signaling, through the generation of PIP2, and LRP6 phosphorylation [82]. In this instance, ARF1 and ARF6 activity was mediated by inactivation of the GTPase activating protein ARFGAP [83].

Aside from its role in promoting structural remodeling conducive to the acquisition of invasive phenotypes, ARF6 activation also has a vital role in propagating Wnt signaling through the formation of signaling endosomes [84,85] (Figure 2). Signaling endosomes are unique endosome populations that serve as a platform for robust and long-lived receptor signal propagation, and a host of cargoes including MAP kinases, various G protein-coupled receptors, and receptor tyrosine kinases (RTKs) such as EGFR, have been identified [86]. In three-dimensional basement membrane cell cultures of epithelial ductal units, ARF6 activation has been shown to promote the formation of signaling endosomes containing growth factor receptors, leading to hyperactive ERK signaling and aberrant epithelial glandular phenotypes resembling certain glandular cancers [84,85]. Wnt3a stimulation of epithelial cysts has been observed to promote ARF6 activity and the subsequent formation of signaling endosomes. Resulting hyperactivation of ERK promotes CK2-dependent phosphorylation of α-catenin to promote the disassembly of cadherin-based adhesions and internalization of adhesion molecules, increasing the cytoplasmic pool of transcriptionally active β-catenin [85]. ERK hyperactivation also leads to increased phosphorylation of the Wnt receptor LRP6, which may allow further amplification of canonical signaling by prompting dissociation of the destruction complex and promoting β-catenin stabilization. These cellular events culminate in filled glandular units reminiscent of pathologies observed in ductal carcinoma in situ (DCIS). Together, these studies advocate a potential role for signaling endosomes in Wnt signal transduction and cancer progression.

### 4.4. Eph and Ephrin Signaling Regulate Small GTPases in Wnt Signaling

Another family of proteins with renewed relevance in cancer, and which regulate Rho GTPases in PCP signaling in development and tissue homeostasis, are ephs and ephrins. Ephrins are membrane-anchored proteins that bind receptor tyrosine kinase eph receptors to stimulate a variety of cell processes mediated by reorganization of the actin cytoskeleton and cell-matrix and cell-cell adhesions, both by forward signaling in the eph-expressing cell and reverse signaling in the ephrin-expressing cell [87,88]. During development, repulsion and attraction cues from ephrin signaling assists in guiding cell movement, attachment, and morphology, mediated by differential activation of Rac, Rho, and Cdc42 [88,89,90,91,92]. Rho exchange factors Ephexin and Vms-RhoGEF preferentially bind to EphA receptors to promote Rho activity, whereas GEFs for Rac1 and Cdc42, Kalirin and Intersectin, associate with EphB receptors [89]. EphrinB1 has been shown to signal through the PCP pathway by interacting with Dvl to activate Rho and JNK, requiring Daam1, to regulate cell migration in Xenopus eye development [93]. An example of ephrin modulation of cell morphology and attachment is seen in melanoma and human embryonic kidney cells, wherein the activation of EphA3 by EphrinA5 activates Rho, causing acto-myosin rearrangement, cell rounding, blebbing, and detachment [94]. Eph-ephrin signaling may function to suppress or promote cancer progression, and many ephrins are overexpressed in cancer [87,95]. An example of the Wnt regulation of differential ephrin signaling in cancer progression is seen in colorectal cancer. In this context, EphB receptors are expressed as Wnt targets, with EphB2 characterized as a tumor suppressor to constrain tumor growth in Apc^Min/+^ mice [96,97], whereas EphB4 promotes cancer progression [98].

### 4.5. Regulation of Small GTPases in Wnt Signal Transduction—The Roles of GAPs and GEFs

Important for regulating the activation of small GTP-binding proteins are their guanine nucleotide exchange factors (GEFs), which facilitate activation by exchanging GTP for GDP, and GTPase activating proteins (GAPs), which promote GTP hydrolysis, rendering the proteins to their inactive, GDP-bound state [99]. Many Wnt signal transduction pathways aided by small GTP-binding proteins, as mentioned in sections above, involve direct targeting of GEFs and GAPs, and a few examples of this regulation are provided.

As mentioned above, there are multiple GAPs and GEFs which regulate ARF activation in Wnt signaling cascades. The activity of the GEF GEP100, stimulated downstream of Wnt5a binding to Fzd4 and LRP6, promoted ARF6 activity and subsequent metastasis in a melanoma xenograft model [46]. ARFGAP, a protein which inactivates ARF1 and ARF6, has been shown to be an important modulator of ARF activity downstream of Wnt3a, regulating LRP6 phosphorylation and the generation of PIP2 [82]. In the case of Rho GTPases, APC has been shown to interact with both APC-stimulated GEF (ASEF), a GEF for Rac1, and IQGAP1, a GAP for both Rac1 and Cdc42. Both ASEF and IQGAP1 are important for regulating directional migration [100]. The activation of Rac1 upon Wnt3a stimulation requires the interaction of the Rac1 GEF Vav2 with p120-catenin, upon the release of catenin from E-cadherin [59]. Additionally, the activation of the planar cell polarity proteins PTK7 (protein tyrosine kinase 7), CELSR (Cadherin EGF LAG seven-pass G-type receptor), and VANG (Van Gogh-like) by Wnts or Fzd receptors may support PCP GTPase signaling by recruiting the Rac1 and Cdc42 GEF PAK, the Rac1 GEF ARHGEF7, and the ARF GAP GIT1 [19,20].

### 4.6. Extracellular Vesicles and Wnt Signaling

In recent years, the study of extracellular vesicles such as exosomes and microvesicles has grown exponentially, largely owing to their importance for the transfer of cargo during normal cellular processes and in disease, and their potential utility as biomarkers [101,102,103,104]. The formation of these vesicles is regulated by a variety of small GTPases [57,78,104,105], and they are enriched with a multitude of cargoes which may facilitate cancer progression, such as nucleic acids and oncogenic receptors which can be transferred to recipient cells to modulate the metastatic niche, and matrix proteases to facilitate cell invasion [101,102,103,104]. The aforementioned ephrin family of signaling molecules has also been characterized as microvesicle cargo [106,107]. Recent work has begun to identify a role for extracellular vesicles in the transfer of Wnts.

Due to their lipid modification, Wnt ligands are highly hydrophobic and traditionally thought to act as short-range signaling molecules [3], constraining their activity to adjacent cells to regulate their potent effects. Wnts have a strong affinity for membranes and the extracellular matrix, with a particular affinity for heparin sulfate proteoglycans [108,109,110,111]. These short range signaling effects of Wnts have been well-described in the literature, identifying important mechanisms that restrict signaling to adjacent cells during certain cellular processes [3,112,113]. There is accumulating evidence that Wnts or downstream Wnt targets may also be modified or packaged into extracellular vesicles to facilitate further distribution, alter gradient formation, or otherwise modulate signaling [13,114,115,116,117,118,119,120,121,122,123,124]. For example, evidence has arisen that modifications which shield the hydrophobic moieties may function as carrier proteins to facilitate long range signaling. In Drosophila, the lipoprotein modification of the Wnt homolog Wingless (Wg) and its packaging into “argosomes” has been shown to be required for its long-range signaling [118]. Additionally, the Wg-interacting lipoprotein Secreted Wingless-Interacting Molecule (SWIM) has been shown to promote solubility of Wg in flies, important for normal wing development [123]. Similarly, mammalian Wnt3a has been shown to be associated with lipoprotein particles [122]. Wnts have been characterized as cargo in extracellular vesicles including exosomes and microvesicles [114,115,116,117,119,120], which may regulate their availability as a ligand. In certain contexts, the packaging of Wnts into vesicles may still mediate short range transfer, such as that which has been demonstrated in synaptic transmission in the Drosophila nervous system [115]. In the context of stem cell renewal, the loading of Wnt3 into embryonic stem cell-derived microvesicles, alongside a complement of other proteins and nucleic acids, has been shown to promote the survival and proliferation of hematopoietic progenitor cells [119]. It has been demonstrated that exosomes may function to remove β-catenin from cells, acting as a mechanism to suppress signaling downstream of Wnt activation [121], and β-catenin has been characterized in exosomes derived from colon cancer cells [106]. Yet another study has shown that carcinoma-associated fibroblasts release extracellular vesicles that stimulate breast cancer cell (BCC) motility and metastasis by mobilizing the non-canonical Wnt/PCP pathway in BCCs [116]. Fibroblast-derived vesicles functioned as a vehicle to tether BCC-produced Wnt11 and facilitate the autocrine signaling of Wnt11 in BCCs. Thus, as an emerging signaling platform, extracellular vesicles could play an important role in facilitating Wnt secretion, transport, and distal signaling.

## 5. Concluding Remarks and Perspectives

Given that Wnt signal transduction is highly influential in regulating GTPase signaling in a variety of normal developmental behaviors and homeostatic tissue maintenance [27,31,58,59], and Wnt signaling is frequently associated with promoting tumor progression [13,22,30,31], the possibility that canonical and non-canonical Wnt signaling cascades may be modulating the GTPase-driven plasticity that cells use to invade through varied environments merits future exploration. Wnt stimulation may provide an additional means for guiding the processes of tissue invasion by specifically targeting key GTPases and/or their effectors.

Though largely unexplored to date, it is possible that the long-range transmission of Wnts, particularly in extracellular vesicles, may play roles in development and disease progression. While exosomes and microvesicles are formed by normal cells, their formation is frequently upregulated in disease states, including cancer [101,102,104]. As has been seen in the case of cancer-associated fibroblasts promoting breast cancer progression through the release of exosomes which modulate Wnt-PCP signaling [116], it is possible that extracellular vesicles may serve as platforms for the dissemination of Wnt proteins and activate canonical or non-canonical pathways in target cells. In development, the highly regulated structure of Wnt gradients is important to constrain Wnt stimulation to isolated cells or cell groups [13], and in some cases, the distribution of Wnt ligands throughout the developing tissue may not be required for the entirety of tissue patterning and development [125]. In the case of cancer, however, the dysregulation of Wnt release which allows for the broadcast of ligand to larger areas may be utilized to promote cell proliferation and motility in tumors or motile cell groups, as well as potentially condition the microenvironment ahead of invasion and colonization. Wnt3a has been shown to function as a chemotactic agent, recruiting cells in a transwell assay, an effect that was distinct from simply upregulating motility [69]. Examples such as this raise the possibility that the deposition of Wnt by extracellular vesicles may promote the directional movement of cells.

Protease deposition from invasive cells is an important mediator of cell invasion which may be accomplished by the release of protease-loaded microvesicles [57,101,102], and matrix metalloproteinase (MMP) upregulation. MMPs are upregulated in response to Wnt stimulation during development and tissue homeostasis [125,126,127,128], required for tissue remodeling, cell migration, and the cleavage of developmental regulatory molecules to mediate their activation [129]. Increased MMP expression is also frequently noted upon Wnt stimulation in tumor cell invasion, including MMP-2 [49], MMP-7 [130], MMP-9 [131], MMP14 [132], and MMP16 [133]. Though currently not investigated, the possibility for an increase in protease expression and its release in extracellular vesicles from invasive tumor cells in response to Wnt stimulation is an area that merits future investigation. Finally, the biomedical community recognizes that extracellular vesicles represent potentially novel avenues for the targeted delivery of customized cargo during disease. These types of research efforts will not only promote the understanding of vesicle-mediated Wnt signaling, but will also advance the exploration of their roles in transmitting other morphogens and signaling molecules, thereby rendering important insights into the biology of Wnt signaling, and ultimately into strategies for intervention in the treatment of Wnt-related diseases.

## References

[1] Mehlen P., Puisieux A. (2006). Metastasis: A question of life or death. Nat. Rev. Cancer.

[2] Hanahan D., Weinberg R.A., Adams J.M., Cory S., Aguirre-Ghiso J.A., Ahmed Z., Bicknell R., Al-Hajj M., Wicha M.S., Benito-Hernandez A. (2011). Hallmarks of Cancer: The Next Generation. Cell.

[3] Clevers H., Loh K.M., Nusse R. (2014). An integral program for tissue renewal and regeneration: Wnt signaling and stem cell control. Science.

[4] Fuerer C., Nusse R., Ten Berge D. (2008). Wnt signalling in development and disease. Max Delbrück Center for Molecular Medicine meeting on Wnt signaling in Development and Disease. EMBO Rep..

[5] Van Amerongen R., Mikels A., Nusse R. (2008). Alternative wnt signaling is initiated by distinct receptors. Sci. Signal..

[6] Willert K., Nusse R. (2012). Wnt Proteins. Cold Spring Harb. Perspect. Biol..

[7] Huang H.C., Klein P.S. (2004). The Frizzled family: Receptors for multiple signal transduction pathways. Genome Biol..

[8] Niehrs C. (2012). The complex world of WNT receptor signalling. Nat. Rev. Mol. Cell Biol..

[9] Mikels A.J., Nusse R. (2006). Wnts as ligands: Processing, secretion and reception. Oncogene.

[10] Green J., Nusse R., van Amerongen R. (2014). The role of Ryk and Ror receptor tyrosine kinases in Wnt signal transduction. Cold Spring Harb. Perspect. Biol..

[11] Clevers H., Nusse R. (2012). Wnt/β-Catenin Signaling and Disease. Cell.

[12] Seifert J.R.K., Mlodzik M. (2007). Frizzled/PCP signalling: A conserved mechanism regulating cell polarity and directed motility. Nat. Rev. Genet..

[13] Clevers H. (2006). Wnt/beta-catenin signaling in development and disease. Cell.

[14] Conacci-Sorrell M., Zhurinsky J., Ben-Ze’ev A. (2002). The cadherin-catenin adhesion system in signaling and cancer. J. Clin. Invest..

[15] Kim S.E., Huang H., Zhao M., Zhang X., Zhang A., Semonov M.V., MacDonald B.T., Zhang X., Garcia A.J., Peng L. (2013). Wnt stabilization of β-catenin reveals principles for morphogen receptor-scaffold assemblies. Science.

[16] Cadigan K.M., Waterman M.L. (2012). TCF/LEFs and Wnt signaling in the nucleus. Cold Spring Harb. Perspect. Biol..

[17] Vlad A., Röhrs S., Klein-Hitpass L., Müller O. (2008). The first five years of the Wnt targetome. Cell. Signal..

[18] Gómez-Orte E., Sáenz-Narciso B., Moreno S., Cabello J. (2013). Multiple functions of the noncanonical Wnt pathway. Trends Genet..

[19] Anastas J.N., Moon R.T. (2013). WNT signalling pathways as therapeutic targets in cancer. Nat. Rev. Cancer.

[20] Wansleeben C., Meijlink F. (2011). The planar cell polarity pathway in vertebrate development. Dev. Dyn..

[21] Wang Y. (2009). Wnt/Planar cell polarity signaling: A new paradigm for cancer therapy. Mol. Cancer Ther..

[22] Jessen J.R. (2009). Noncanonical Wnt signaling in tumor progression and metastasis. Zebrafish.

[23] Siar C.H., Nagatsuka H., Han P.P., Buery R.R., Tsujigiwa H., Nakano K., Ng K.H., Kawakami T. (2012). Differential expression of canonical and non-canonical Wnt ligands in ameloblastoma. J. Oral Pathol. Med..

[24] Gong Y., Mo C., Fraser S.E. (2004). Planar cell polarity signalling controls cell division orientation during zebrafish gastrulation. Nature.

[25] Ueno N., Greene N.D.E. (2003). Planar cell polarity genes and neural tube closure. Birth Defects Res. C. Embryo Today.

[26] Marshall W.F. (2010). Cilia self-organize in response to planar cell polarity and flow. Nat. Cell Biol..

[27] Mayor R., Theveneau E. (2014). The role of the non-canonical Wnt-planar cell polarity pathway in neural crest migration. Biochem. J..

[28] Wallingford J.B., Rowning B.A., Vogeli K.M., Rothbächer U., Fraser S.E., Harland R.M. (2000). Dishevelled controls cell polarity during Xenopus gastrulation. Nature.

[29] Gilbert S.F. (2000). The Neural Crest. Developmental Biology.

[30] Giles R.H., van Es J.H., Clevers H. (2003). Caught up in a Wnt storm: Wnt signaling in cancer. Biochim. Biophys. Acta.

[31] Ishida-Takagishi M., Enomoto A., Asai N., Ushida K., Watanabe T., Hashimoto T., Kato T., Weng L., Matsumoto S., Asai M. (2012). The Dishevelled-associating protein Daple controls the non-canonical Wnt/Rac pathway and cell motility. Nat. Commun..

[32] Nusse R., Varmus H.E. (1982). Many tumors induced by the mouse mammary tumor virus contain a provirus integrated in the same region of the host genome. Cell.

[33] Nusse R., Brown A., Papkoff J., Scambler P., Shackleford G., McMahon A., Moon R., Varmus H. (1991). A new nomenclature for *int*-1 and related genes: The Wnt gene family. Cell.

[34] Kinzler K.W., Nilbert M.C., Su L.K., Vogelstein B., Bryan T.M., Levy D.B., Smith K.J., Preisinger A.C., Hedge P., McKechnie D. (1991). Identification of FAP locus genes from chromosome 5q21. Science.

[35] Groden J., Thliveris A., Samowitz W., Carlson M., Gelbert L., Albertsen H., Joslyn G., Stevens J., Spirio L., Robertson M. (1991). Identification and characterization of the familial adenomatous polyposis coli gene. Cell.

[36] Fearnhead N.S. (2001). The ABC of APC. Hum. Mol. Genet..

[37] Kwong L.N., Dove W.F. (2009). APC and its modifiers in colon cancer. Adv. Exp. Med. Biol..

[38] Furuuchi K., Tada M., Yamada H., Kataoka A., Furuuchi N., Hamada J., Takahashi M., Todo S., Moriuchi T. (2000). Somatic mutations of the APC gene in primary breast cancers. Am. J. Pathol..

[39] Ohgaki H., Kros J.M., Okamoto Y., Gaspert A., Huang H., Kurrer M.O. (2004). APC mutations are infrequent but present in human lung cancer. Cancer Lett..

[40] Nakatsuru S., Yanagisawa A., Ichii S., Tahara E., Kato Y., Nakamura Y., Horii A. (1992). Somatic mutation of the APC gene in gastric cancer: Frequent mutations in very well differentiated adenocarcinoma and signet-ring cell carcinoma. Hum. Mol. Genet..

[41] Arend R.C., Londoño-Joshi A.I., Straughn J.M., Buchsbaum D.J. (2013). The Wnt/β-catenin pathway in ovarian cancer: A review. Gynecol. Oncol..

[42] Miyoshi Y., Iwao K., Nagasawa Y., Aihara T., Sasaki Y., Imaoka S., Murata M., Shimano T., Nakamura Y. (1998). Activation of the β-catenin gene in primary hepatocellular carcinomas by somatic alterations involving exon 3. Cancer Res..

[43] Mazzoni S.M., Fearon E.R. (2014). AXIN1 and AXIN2 variants in gastrointestinal cancers. Cancer Lett..

[44] Da Forno P.D., Pringle J.H., Hutchinson P., Osborn J., Huang Q., Potter L., Hancox R.A., Fletcher A., Saldanha G.S. (2008). WNT5A expression increases during melanoma progression and correlates with outcome. Clin. Cancer Res..

[45] Kurayoshi M., Oue N., Yamamoto H., Kishida M., Inoue A., Asahara T., Yasui W., Kikuchi A. (2006). Expression of Wnt-5a is correlated with aggressiveness of gastric cancer by stimulating cell migration and invasion. Cancer Res..

[46] Grossmann A.H., Yoo J.H., Clancy J., Sorensen L.K., Sedgwick A., Tong Z., Ostanin K., Rogers A., Grossmann K.F., Tripp S.R. (2013). The small GTPase ARF6 stimulates β-catenin transcriptional activity during Wnt5a-mediated melanoma invasion and metastasis. Sci. Signal..

[47] Bo H., Gao L., Chen Y., Zhang J., Zhu M. (2016). Upregulation of the expression of Wnt5a promotes the proliferation of pancreatic cancer cells in vitro and in a nude mouse model. Mol. Med. Rep..

[48] Weeraratna A.T., Jiang Y., Hostetter G., Rosenblatt K., Duray P., Bittner M., Trent J.M. (2002). Wnt5a signaling directly affects cell motility and invasion of metastatic melanoma. Cancer Cell.

[49] Kamino M., Kishida M., Kibe T., Ikoma K., Iijima M., Hirano H., Tokudome M., Chen L., Koriyama C., Yamada K. (2011). Wnt-5a signaling is correlated with infiltrative activity in human glioma by inducing cellular migration and MMP-2. Cancer Sci..

[50] Leris A.C.A., Roberts T.R., Jiang W.G., Newbold R.F., Mokbel K. (2005). WNT5A expression in human breast cancer. Anticancer Res..

[51] Jonsson M., Dejmek J., Bendahl P.O., Andersson T. (2002). loss of Wnt-5a protein is associated with early relapse in invasive ductal breast carcinomas. Cancer Res..

[52] Dejmek J., Leandersson K., Manjer J., Bjartell A., Emdin S.O., Vogel W.F., Landberg G., Andersson T. (2005). expression and signaling activity of Wnt-5a/discoidin domain receptor-1 and Syk plays distinct but decisive roles in breast cancer patient survival. Clin. Cancer Res..

[53] Schadendorf D., Fisher D.E., Garbe C., Gershenwald J.E., Grob J.J., Halpern A., Herlyn M., Marchetti M.A., McArthur G., Ribas A. (2015). Melanoma. Nat. Rev. Dis. Prim..

[54] Gaggioli C., Sahai E. (2007). Melanoma invasion—Current knowledge and future directions. Pigment Cell Res..

[55] Sanz-Moreno V., Gadea G., Ahn J., Paterson H., Marra P., Pinner S., Sahai E., Marshall C.J. (2008). Rac activation and inactivation control plasticity of tumor cell movement. Cell.

[56] Friedl P., Wolf K. (2010). Plasticity of cell migration: A multiscale tuning model. J. Cell Biol..

[57] Sedgwick A.E., Clancy J.W., Olivia Balmert M., D’Souza-Schorey C. (2015). Extracellular microvesicles and invadopodia mediate non-overlapping modes of tumor cell invasion. Sci. Rep..

[58] Schlessinger K., Hall A., Tolwinski N. (2009). Wnt signaling pathways meet Rho GTPases. Genes Dev..

[59] Valls G., Codina M., Miller R.K., del Valle-Pérez B., Vinyoles M., Caelles C., McCrea P.D., García de Herreros A., Duñach M. (2012). Upon Wnt stimulation, Rac1 activation requires Rac1 and Vav2 binding to p120-catenin. J. Cell Sci..

[60] Wu X., Tu X., Joeng K.S., Hilton M.J., Williams D.A., Long F. (2008). Rac1 activation controls nuclear localization of beta-catenin during canonical Wnt signaling. Cell.

[61] Fukukawa C., Nagayama S., Tsunoda T., Toguchida J., Nakamura Y., Katagiri T. (2009). Activation of the non-canonical Dvl-Rac1-JNK pathway by Frizzled homologue 10 in human synovial sarcoma. Oncogene.

[62] Matthews H.K., Marchant L., Carmona-Fontaine C., Kuriyama S., Larraín J., Holt M.R., Parsons M., Mayor R. (2008). Directional migration of neural crest cells in vivo is regulated by Syndecan-4/Rac1 and non-canonical Wnt signaling/RhoA. Development.

[63] Otto A., Collins-Hooper H., Patel A., Dash P.R., Patel K. (2011). Adult skeletal muscle stem cell migration is mediated by a blebbing/amoeboid mechanism. Rejuvenation Res..

[64] Theveneau E., Mayor R. (2012). Neural crest delamination and migration: From epithelium-to-mesenchyme transition to collective cell migration. Dev. Biol..

[65] Parri M., Chiarugi P. (2010). Rac and Rho GTPases in cancer cell motility control. Cell Commun. Signal..

[66] Weiser D.C., Row R.H., Kimelman D. (2009). Rho-regulated myosin phosphatase establishes the level of protrusive activity required for cell movements during zebrafish gastrulation. Development.

[67] Bishop A.L., Hall A. (2000). Rho GTPases and their effector proteins. Biochem. J..

[68] Rodrigues P., Macaya I., Bazzocco S., Mazzolini R., Andretta E., Dopeso H., Mateo-Lozano S., Bilić J., Cartón-García F., Nieto R. (2014). RHOA inactivation enhances Wnt signalling and promotes colorectal cancer. Nat. Commun..

[69] Endo Y., Wolf V., Muraiso K., Kamijo K., Soon L., Uren A., Barshishat-Küpper M., Rubin J.S. (2005). Wnt-3a-dependent cell motility involves RhoA activation and is specifically regulated by dishevelled-2. J. Biol. Chem..

[70] Iwai S., Yonekawa A., Harada C., Hamada M., Katagiri W., Nakazawa M., Yura Y. (2010). Involvement of the Wnt-β-catenin pathway in invasion and migration of oral squamous carcinoma cells. Int. J. Oncol..

[71] Toyama T., Lee H.C., Koga H., Wands J.R., Kim M. (2010). Noncanonical Wnt11 inhibits hepatocellular carcinoma cell proliferation and migration. Mol. Cancer Res..

[72] D’Souza-Schorey C., Chavrier P. (2006). ARF proteins: Roles in membrane traffic and beyond. Nat. Rev. Mol. Cell Biol..

[73] Suzuki T., Kanai Y., Hara T., Sasaki J., Sasaki T., Kohara M., Maehama T., Taya C., Shitara H., Yonekawa H. (2006). Crucial role of the small GTPase ARF6 in hepatic cord formation during liver development. Mol. Cell. Biol..

[74] Knizhnik A.V., Kovaleva O.B., Laktionov K.K., Mochal’nikova V.V., Komel’kov A.V., Chevkina E.M., Zborovskaia I.B. (2011). ARF6, RalA and BIRC5 protein expression in non small cell lung cancer. Mol. Biol..

[75] Hashimoto S., Onodera Y., Hashimoto A., Tanaka M., Hamaguchi M., Yamada A., Sabe H. (2004). Requirement for ARF6 in breast cancer invasive activities. Proc. Natl. Acad. Sci. USA.

[76] Morgan C., Lewis P.D., Hopkins L., Burnell S., Kynaston H., Doak S.H. (2015). Increased expression of ARF GTPases in prostate cancer tissue. SpringerPlus.

[77] Bauer K.M., Hummon A.B. (2012). Effects of the miR-143/-145 microRNA cluster on the colon cancer proteome and transcriptome. J. Proteome Res..

[78] Muralidharan-Chari V., Clancy J., Plou C., Romao M., Chavrier P., Raposo G., D’Souza-Schorey C. (2009). ARF6-regulated shedding of tumor cell-derived plasma membrane microvesicles. Curr. Biol..

[79] Muralidharan-Chari V., Hoover H., Clancy J., Schweitzer J., Suckow M.A., Schroeder V., Castellino F.J., Schorey J.S., D’Souza-Schorey C. (2009). ADP-ribosylation factor 6 regulates tumorigenic and invasive properties in vivo. Cancer Res..

[80] Davies J.P., Ioannou Y.A. (2000). Topological analysis of Niemann-Pick C1 protein reveals that the membrane orientation of the putative sterol-sensing domain is identical to those of 3-hydroxy-3-methylglutaryl-CoA reductase and sterol regulatory element binding protein cleavage-activating. J. Biol. Chem..

[81] Tague S.E., Muralidharan V., D’Souza-Schorey C. (2004). ADP-ribosylation factor 6 regulates tumor cell invasion through the activation of the MEK/ERK signaling pathway. Proc. Natl. Acad. Sci. USA.

[82] Kim W., Kim S.Y., Kim T., Kim M., Bae D.J., Choi H.I., Kim I.S., Jho E. (2013). ADP-ribosylation factors 1 and 6 regulate Wnt/β-catenin signaling via control of LRP6 phosphorylation. Oncogene.

[83] Zhang Q., Major M.B., Takanashi S., Camp N.D., Nishiya N., Peters E.C., Ginsberg M.H., Jian X., Randazzo P.A., Schultz P.G. (2007). Small-molecule synergist of the Wnt/beta-catenin signaling pathway. Proc. Natl. Acad. Sci. USA.

[84] Tushir J.S., Clancy J., Warren A., Wrobel C., Brugge J.S., D’Souza-Schorey C. (2010). Unregulated ARF6 activation in epithelial cysts generates hyperactive signaling endosomes and disrupts morphogenesis. Mol. Biol. Cell.

[85] Pellon-Cardenas O., Clancy J., Uwimpuhwe H., D’Souza-Schorey C. (2013). ARF6-regulated endocytosis of growth factor receptors links cadherin-based adhesion to canonical Wnt signaling in epithelia. Mol. Cell. Biol..

[86] Sorkin A., von Zastrow M. (2009). Endocytosis and signalling: Intertwining molecular networks. Nat. Rev. Mol. Cell Biol..

[87] Pasquale E.B. (2010). Eph receptors and ephrins in cancer: Bidirectional signalling and beyond. Nat. Rev. Cancer.

[88] Klein R. (2012). Eph/ephrin signalling during development. Development.

[89] Noren N.K., Pasquale E.B. (2004). Eph receptor-ephrin bidirectional signals that target Ras and Rho proteins. Cell. Signal..

[90] Park I., Lee H.S. (2015). EphB/ephrinB signaling in cell adhesion and migration. Mol. Cells.

[91] Park E.C., Cho G.S., Kim G.H., Choi S.C., Han J.K. (2011). The involvement of Eph-Ephrin signaling in tissue separation and convergence during Xenopus gastrulation movements. Dev. Biol..

[92] Gale N.W., Yancopoulos G.D. (1997). Ephrins and their receptors: A repulsive topic?. Cell Tissue Res..

[93] Lee H.S., Bong Y.S., Moore K.B., Soria K., Moody S.A., Daar I.O. (2006). Dishevelled mediates ephrinB1 signalling in the eye field through the planar cell polarity pathway. Nat. Cell Biol..

[94] Lawrenson I.D., Wimmer-Kleikamp S.H., Lock P., Schoenwaelder S.M., Down M., Boyd A.W., Alewood P.F., Lackmann M. (2002). Ephrin-A5 induces rounding, blebbing and de-adhesion of EphA3-expressing 293T and melanoma cells by CrkII and Rho-mediated signalling. J. Cell Sci..

[95] Surawska H., Ma P.C., Salgia R. (2004). The role of ephrins and Eph receptors in cancer. Cytokine Growth Factor Rev..

[96] Cortina C., Palomo-Ponce S., Iglesias M., Fernández-Masip J.L., Vivancos A., Whissell G., Humà M., Peiró N., Gallego L., Jonkheer S. (2007). EphB-ephrin-B interactions suppress colorectal cancer progression by compartmentalizing tumor cells. Nat. Genet..

[97] Clevers H., Batlle E. (2006). EphB/EphrinB receptors and Wnt signaling in colorectal cancer. Cancer Res..

[98] Kumar S.R., Scehnet J.S., Ley E.J., Singh J., Krasnoperov V., Liu R., Manchanda P.K., Ladner R.D., Hawes D., Weaver F.A. (2009). Preferential induction of EphB4 over EphB2 and its implication in colorectal cancer progression. Cancer Res..

[99] Bos J.L., Rehmann H., Wittinghofer A. (2007). GEFs and GAPs: Critical elements in the control of small G proteins. Cell.

[100] Akiyama T., Kawasaki Y. (2006). Wnt signalling and the actin cytoskeleton. Oncogene.

[101] Muralidharan-Chari V., Clancy J.W., Sedgwick A., D’Souza-Schorey C. (2010). Microvesicles: Mediators of extracellular communication during cancer progression. J. Cell Sci..

[102] Van Doormaal F.F., Kleinjan A., di Nisio M., Büller H.R., Nieuwland R. (2009). Cell-derived microvesicles and cancer. Neth. J. Med..

[103] Lee T.H., D’Asti E., Magnus N., Al-Nedawi K., Meehan B., Rak J. (2011). Microvesicles as mediators of intercellular communication in cancer—the emerging science of cellular “debris”. Semin. Immunopathol..

[104] D’Souza-Schorey C., Clancy J.W. (2012). Tumor-derived microvesicles: Shedding light on novel microenvironment modulators and prospective cancer biomarkers. Genes Dev..

[105] Li B., Antonyak M.A., Zhang J., Cerione R.A. (2012). RhoA triggers a specific signaling pathway that generates transforming microvesicles in cancer cells. Oncogene.

[106] Tauro B.J., Greening D.W., Mathias R.A., Ji H., Mathivanan S., Scott A.M., Simpson R.J. (2012). Comparison of ultracentrifugation, density gradient separation, and immunoaffinity capture methods for isolating human colon cancer cell line LIM1863-derived exosomes. Methods.

[107] Sandvig K., Llorente A. (2012). Proteomic analysis of microvesicles released by the human prostate cancer cell line PC-3. Mol. Cell. Proteomics.

[108] Papkoff J., Schryver B. (1990). Secreted int-1 protein is associated with the cell surface. Mol. Cell. Biol..

[109] Papkoff J., Brown A.M., Varmus H.E. (1987). The int-1 proto-oncogene products are glycoproteins that appear to enter the secretory pathway. Mol. Cell. Biol..

[110] Bradley R.S., Brown A.M. (1990). The proto-oncogene int-1 encodes a secreted protein associated with the extracellular matrix. EMBO J..

[111] Fuerer C., Habib S.J., Nusse R. (2010). A study on the interactions between heparan sulfate proteoglycans and Wnt proteins. Dev. Dyn..

[112] Farin H.F., Jordens I., Mosa M.H., Basak O., Korving J., Tauriello D.V.F., de Punder K., Angers S., Peters P.J., Maurice M.M. (2016). Visualization of a short-range Wnt gradient in the intestinal stem-cell niche. Nature.

[113] Habib S.J., Chen B.C., Tsai F.C., Anastassiadis K., Meyer T., Betzig E., Nusse R., Neumüller R.A., Knoblich J.A., Werts A.D. (2013). A localized Wnt signal orients asymmetric stem cell division in vitro. Science.

[114] Zhang L., Wrana J.L. (2014). The emerging role of exosomes in Wnt secretion and transport. Curr. Opin. Genet. Dev..

[115] Korkut C., Ataman B., Ramachandran P., Ashley J., Barria R., Gherbesi N., Budnik V. (2009). Trans-synaptic transmission of vesicular Wnt signals through Evi/Wntless. Cell.

[116] Luga V., Zhang L., Viloria-Petit A.M., Ogunjimi A.A., Inanlou M.R., Chiu E., Buchanan M., Hosein A.N., Basik M., Wrana J.L. (2012). Exosomes mediate stromal mobilization of autocrine Wnt-PCP signaling in breast cancer cell migration. Cell.

[117] Coudreuse D., Korswagen H.C. (2007). The making of Wnt: New insights into Wnt maturation, sorting and secretion. Development.

[118] Panáková D., Sprong H., Marois E., Thiele C., Eaton S. (2005). Lipoprotein particles are required for Hedgehog and Wingless signalling. Nature.

[119] Ratajczak J., Miekus K., Kucia M., Zhang J., Reca R., Dvorak P., Ratajczak M.Z. (2006). Embryonic stem cell-derived microvesicles reprogram hematopoietic progenitors: Evidence for horizontal transfer of mRNA and protein delivery. Leukemia.

[120] Gross J.C., Chaudhary V., Bartscherer K., Boutros M. (2012). Active Wnt proteins are secreted on exosomes. Nat. Cell Biol..

[121] Chairoungdua A., Smith D.L., Pochard P., Hull M., Caplan M.J. (2010). Exosome release of β-catenin: A novel mechanism that antagonizes Wnt signaling. J. Cell Biol..

[122] Neumann S., Coudreuse D.Y.M., van der Westhuyzen D.R., Eckhardt E.R.M., Korswagen H.C., Schmitz G., Sprong H. (2009). Mammalian Wnt3a is released on lipoprotein particles. Traffic.

[123] Mulligan K.A., Fuerer C., Ching W., Fish M., Willert K., Nusse R. (2012). Secreted Wingless-interacting molecule (Swim) promotes long-range signaling by maintaining Wingless solubility. Proc. Natl. Acad. Sci. USA.

[124] Hausmann G., Bänziger C., Basler K. (2007). Helping Wingless take flight: How Wnt proteins are secreted. Nat. Rev. Mol. Cell Biol..

[125] Alexandre C., Baena-Lopez A., Vincent J.P. (2013). Patterning and growth control by membrane-tethered Wingless. Nature.

[126] Wu B., Crampton S.P., Hughes C.C.W. (2007). Wnt signaling induces matrix metalloproteinase expression and regulates T cell transmigration. Immunity.

[127] Ingraham C.A., Park G.C., Makarenkova H.P., Crossin K.L. (2011). Matrix metalloproteinase (MMP)-9 induced by Wnt signaling increases the proliferation and migration of embryonic neural stem cells at low O2 levels. J. Biol. Chem..

[128] Lyu J., Joo C.K. (2005). Wnt-7a up-regulates matrix metalloproteinase-12 expression and promotes cell proliferation in corneal epithelial cells during wound healing. J. Biol. Chem..

[129] Vu T.H. (2000). Matrix metalloproteinases: Effectors of development and normal physiology. Genes Dev..

[130] Pukrop T., Klemm F., Hagemann T., Gradl D., Schulz M., Siemes S., Trümper L., Binder C. (2006). Wnt 5a signaling is critical for macrophage-induced invasion of breast cancer cell lines. Proc. Natl. Acad. Sci. USA.

[131] Lee M.A., Park J.H., Rhyu S.Y., Oh S.T., Kang W.K., Kim H.N. (2014). Wnt3a expression is associated with MMP-9 expression in primary tumor and metastatic site in recurrent or stage IV colorectal cancer. BMC Cancer.

[132] Takahashi M., Tsunoda T., Seiki M., Nakamura Y., Furukawa Y. (2002). Identification of membrane-type matrix metalloproteinase-1 as a target of the β-catenin/TCF4 complex in human colorectal cancers. Oncogene.

[133] Lowy A.M., Clements W.M., Bishop J., Kong L., Bonney T., Sisco K., Aronow B., Fenoglio-Preiser C., Groden J. (2006). β-Catenin/Wnt signaling regulates expression of the membrane type 3 matrix metalloproteinase in gastric cancer. Cancer Res..

